# The Links of Ghrelin to Incretins, Insulin, Glucagon, and Leptin After Bariatric Surgery

**DOI:** 10.3389/fgene.2021.612501

**Published:** 2021-04-20

**Authors:** Daria Skuratovskaia, Maria Vulf, Nataliya Chasovskikh, Aleksandra Komar, Elena Kirienkova, Egor Shunkin, Pavel Zatolokin, Larisa Litvinova

**Affiliations:** ^1^Center for Immunology and Cellular Biotechnology, Immanuel Kant Baltic Federal University, Kaliningrad, Russia; ^2^Department of Medical and Biological Cybernetics, Siberian State Medical University, Tomsk, Russia

**Keywords:** gene annotation, type 2 diabetes mellitus, bariatric surgery, GHSR, GIPR, GLP-1r, adipokines, obesity

## Abstract

Type 2 diabetes mellitus (T2DM) is one of the most prominent and socially significant problems. The present study aimed to identify the mechanisms of interaction of critical regulators of carbohydrate metabolism using bioinformatics and experimental methods and to assess their influence on the development of T2DM. We conducted an *in silico* search for the relationship of hormones and adipokines and performed functional annotation of the receptors for ghrelin and incretins. Hormones and adipokines were assessed in the plasma of obese patients with and without T2DM as well as after laparoscopic sleeve gastrectomy (LSG) and Roux-en-Y gastric bypass (RYGB) surgeries. Incretin- and ghrelin-associated functions and metabolic processes were discovered. Low ghrelin levels were observed in obese patients without T2DM compared with healthy volunteers and the other groups. The highest ghrelin levels were observed in obese patients with T2DM. This defense mechanism against insulin resistance could be realized through the receptors G-protein-coupled receptor (GPCR), growth hormone secretagogue receptor (GHSR), and growth hormone-releasing hormone receptor (GHRHR). These receptors are associated with proliferative, inflammatory, and neurohumoral signaling pathways and regulate responses to nutrient intake. Signaling through the GPCR class unites ghrelin, glucagon, glucose-dependent insulinotropic polypeptide (GIP), and glucagon-like peptide (GLP)-1. Ghrelin impairs carbohydrate and lipid metabolism in obese patients. Ghrelin is associated with elevated plasma levels of insulin, glucagon, and leptin. Specific activation of receptors and modulation by posttranslational modifications of ghrelin can control IR’s development in obesity, which is a promising area for research.

## Introduction

Type 2 diabetes mellitus (T2DM) is one of the most prominent and socially significant problems (Dedov et al., 2017; Statistics About Diabetes | ADA, 2020). T2DM and associated diseases, in particular, abdominal obesity, occupy a leading position among the causes of mortality in the population (WHO | Raised fasting blood glucose, 2020). T2DM is characterized by the impaired metabolic response of insulin-dependent tissue [e.g., muscle, adipose tissue (AT), liver] to insulin, which leads to an increase in its concentration in human plasma (Finan et al., 2013). It is known that gastroduodenal zone hormones and mediators of AT, namely, adipokines, regulate carbohydrate metabolism components and play an important role in the pathogenesis of IR in T2DM (Finan et al., 2013; Vejrazkova et al., 2017). Different parts of the intestine secrete many hormones, and the key role belongs to incretin glucose-dependent insulinotropic polypeptide (GIP) and glucagon-like peptide-1 (GLP-1) (Skow et al., 2016; Brandt et al., 2018).

The main function of incretins is to stimulate insulin secretion by the pancreatic β-cells of the islets of Langerhans (Yabe and Seino, 2011; Campbell et al., 2016). GLP-1 promotes the normalization of carbohydrate metabolism and decreases body mass index (BMI) (Hong et al., 2016), while GIP has multidirectional effects on carbohydrate metabolism. The role of GIP in incretins is still controversial (Finan et al., 2013). It has been demonstrated that incretins are interrelated with the regulation of leptin and ghrelin production, the main modulators of carbohydrate metabolism (Kim and Egan, 2008; Karim et al., 2015; Ronveaux et al., 2015). Ghrelin is an orexigenic hormone that increases ingestion by activating agouti-related peptide (AgRP)/neuropeptide Y (NPY) neurons (Yanagi et al., 2018). Fasting plasma ghrelin level increases, then ghrelin activates gluconeogenesis in the liver, suppresses insulin production, and maintains glucose levels within control parameters. Postprandial hormone production decreases when there is a glucose-stimulated increase in insulin secretion.

In addition to metabolic functions, leptin is a pleiotropic inflammatory mediator and modulates glucose homeostasis and insulin release by reducing glucagon secretion (Hong et al., 2016; Vejrazkova et al., 2017). Glucagon stimulates glucose production in the liver, preventing hypoglycemia under normal physiological conditions; hyperglucagonemia is an indicator of T2DM (Vejrazkova et al., 2017; Brandt et al., 2018). It has been established that resistin also promotes the development of IR (Stofkova, 2010; Vejrazkova et al., 2017). Visfatin has pro-inflammatory and immunomodulatory properties and has insulin-sensitizing and insulin-mimetic effects. Thus, visfatin is of interest as a possible target for modulating blood glucose (Stofkova, 2010; Vejrazkova et al., 2017).

Incretin-stimulated insulin secretion accounts for approximately 50% of the total insulin production (Kim and Egan, 2008). Insulin biosynthesis and secretion are closely related to incretin receptors (Kim and Egan, 2008). In patients with T2DM, the sensitivity of cells to insulin is reduced, and glucose-dependent secretion of insulin is impaired (Nielsen et al., 2015). It has been established that in patients with T2DM, the absence and decrease in response to incretin therapy may be associated with dysregulation of expression or defects in incretin receptors (Yabe and Seino, 2013). Therefore, incretins play an important role in the regulation of insulin production. Consequently, studies of the causes of impaired secretion of incretins and decreased insulin-dependent receptor sensitivity in T2DM are relevant.

The present study aimed to identify the mechanisms of interaction of critical regulators of carbohydrate metabolism using bioinformatics and experimental methods, and to assess their influence on the development of T2DM complicated by obesity.

## Materials and Methods

### Experimental Research Methods

The study included 225 obese patients. Of these, 113 obese patients had T2DM (45.18 ± 8.29 years; 45.69 ± 10.51 kg/m^2^; 46 men and 67 women), and 115 obese patients did not have carbohydrate metabolism disorders (46.41 ± 9.3 years; 46.31 ± 7.56 kg/m^2^; 41 men and 74 women). The presence of arterial hypertension was noted in 43% of patients. Obese patients with T2DM underwent surgery with two types of surgical treatment: laparoscopic sleeve gastrectomy (LSG) (48.53 ± 6.13 years; 40.59 ± 6.55 kg/m^2^) and Roux-en-Y gastric bypass (RYGB) (46.08 ± 10.63 years; 33.52 ± 6.08 kg/m^2^). The results of these patients were recorded 6 months after surgery. The control group of healthy volunteers included 102 apparently healthy donors with normal anthropometric and biochemical parameters (39.3 ± 6.51 years; 22.82 ± 2.18 kg/m^2^; 61 men and 41 women). Venous blood was obtained before and 60 min after the test breakfast. In the test breakfast, the protein content was 9.1 g, the carbohydrate content was 88.1 g, and the fat content was 10.6 g. In 45% of the patients, essential hypertension was diagnosed according to the classification of arterial hypertension by the arterial pressure level (EHS/ESC 2003–2013).

All the study participants provided informed consent to participate in the research study. The study was carried out according to the World Medical Association (WMA) Declaration of Helsinki (2000) and the Protocol to the Convention on Human Rights and Biomedicine (1999). The Local Ethics Committee approved the study protocol of the Innovation Park of the Immanuel Kant Baltic Federal University (protocol no. 4 from October 23, 2013).

The analysis of biochemical parameters in the blood serum was carried out on a Furuno CA-180 automatic biochemical analyzer (Furuno Electric Company, Japan) using DiaSys test systems (DiaSys Diagnostic Systems, Germany). Plasma hormone levels were assessed by flow fluorimetry (Bio-Plex Protein Assay System, Bio-Rad, United States) using commercial test systems (Bio-Plex Pro Human Diabetes 10-Plex Assay, Bio-Rad, United States). In obese patients and healthy donors, the concentrations of mediators (hormones: ghrelin, GIP, GLP-1, insulin, C-peptide, and glucagon) and the mediator SERPINE1 (PAI-1) and adipokines (resistin, leptin, and visfatin) were assessed in plasma.

**FIGURE 1:**
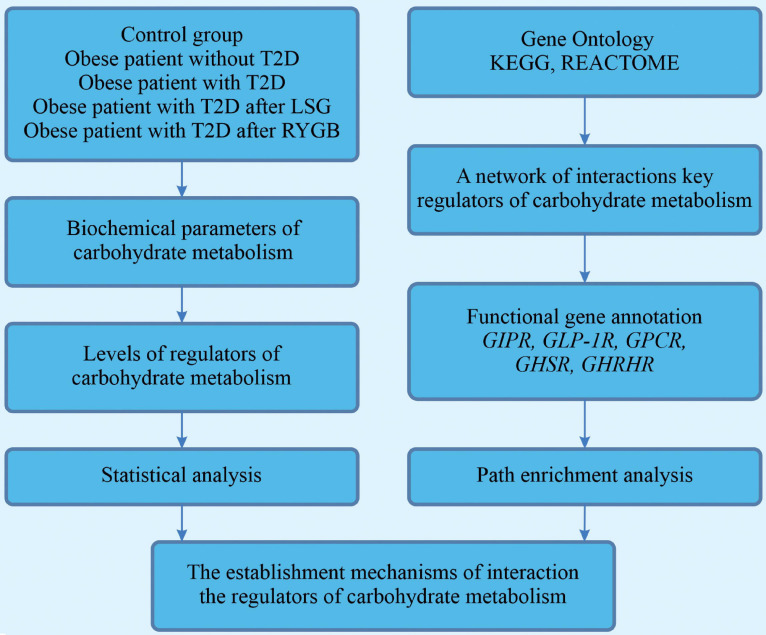
A block-diagram of the research.

A block diagram of the research is presented in Figure 1.

### Bioinformatic Research Methods

Interactions of the studied proteins (nodes: proteins; edges: cooperations between them) were evaluated using the Cytoscape version 3.2.1 (United States) network.

Initial data on protein–protein interactions were obtained from the Human Protein Reference Database (HPRD). Protein–protein interactions from the HPRDs were used to construct a network of interactions for the proteins under study (Keshava Prasad et al., 2009). In the resulting network, proteins from the HPRDs interacting with these proteins and common bonds were identified. All studied proteins were applied to the protein–protein interaction HPRD network and then extracted together with the associated proteins.

The functional annotation of the gastric inhibitory polypeptide receptor (GIPR) and glucagon-like peptide-1 receptor (GLP-1R) genes and the analysis of their representation in the signaling and metabolic pathways were carried out using the algorithm implemented in the ClueGO Cytoscape version 3.2.1 plugin (Bindea et al., 2009) based on the use of the hypergeometric test (*p* < 0.05). The value of the kappa statistic, reflecting the functional relationships between genes, was set at 0.4. The functional characterization of genes was carried out based on the terminology of Gene Ontology within the categories of “biological process” and “molecular function” (Ashburner et al., 2000; Rivals et al., 2007). In this study, the functions were described from the third to the eighth levels of the hierarchy. The representation of genes in signaling and metabolic pathways was determined using the KEGG (Kyoto Encyclopedia of Genes and Genomes) pathway and Reactome pathway analyses.

### Statistical Analysis of Experimental Data

The normal distribution of quantitative indicators was checked using the Kolmogorov–Smirnov test. If the normal law of distributing a feature in the studied samples was consistent, the hypothesis about the average sample values’ equality was tested using Student’s *t* test. If the data distribution did not obey the normal distribution law, further assessment of the sample differences was calculated using the non-parametric Mann–Whitney test for pairwise comparisons. According to the Spearman method, a relationship between the studied parameters was carried out using correlation analysis. Differences were considered significant at a significance level of *p* < 0.05. Statistical processing of the obtained results was carried out using the R statistical software (version 3.3.1).

## Results

### Ghrelin Is Related to Insulin

Body mass index and biochemical parameters of carbohydrate metabolism (glucose and insulin) and lipid metabolism (cholesterol, triglycerides, HDL, and LDL) were measured in obese patients. Predictably, we observed a disturbance in carbohydrate and lipid metabolism parameters in obese patients with T2DM (Supplementary Table 1). We investigated a comparison group of obese patients without T2DM to look for the involvement of mediators in maintaining normal glucose levels. These obese patients had a high BMI, but carbohydrate and lipid metabolism were within normal limits, and hormone levels were comparable or lower than healthy volunteers. We did not find any sex differences in parameters between groups.

**FIGURE 2:**
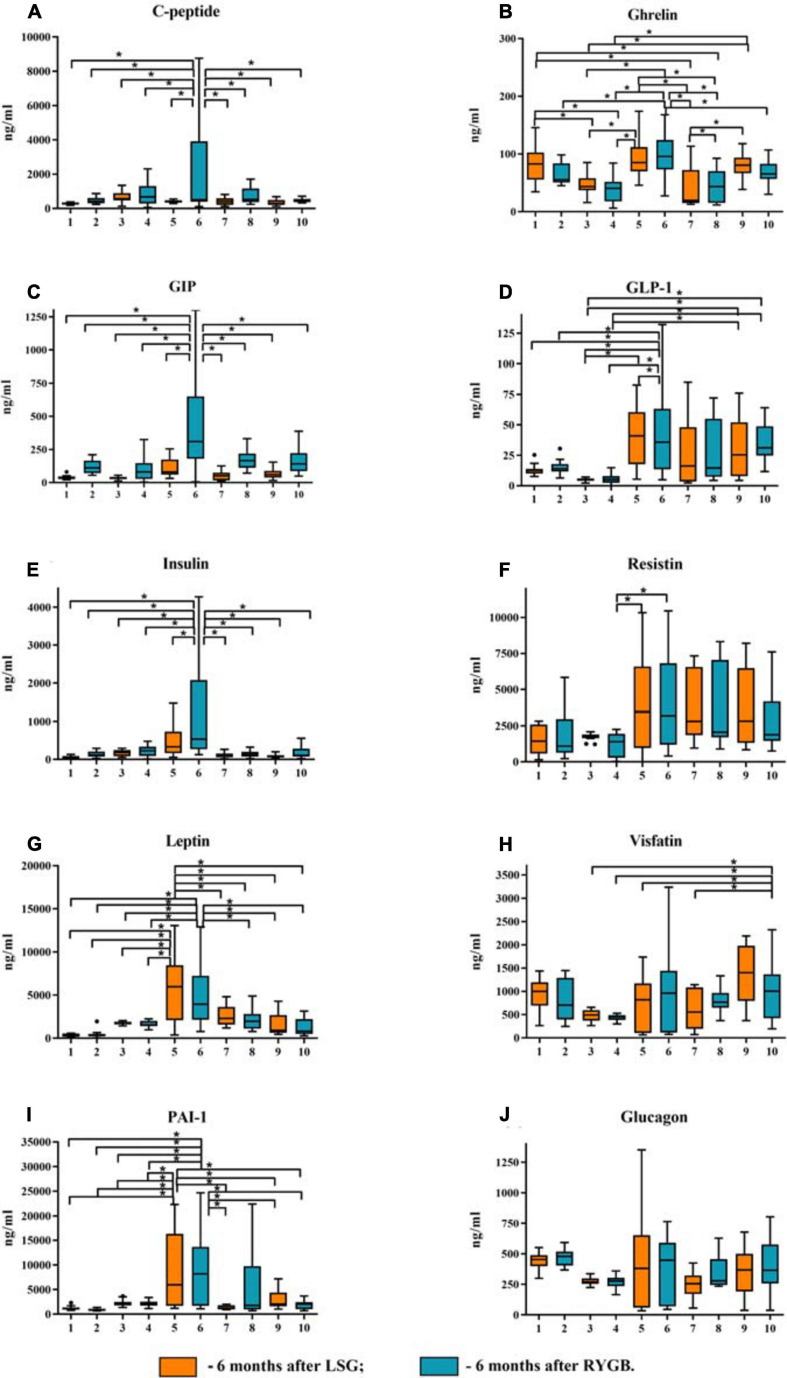
Plasma levels of the studied mediators in obese patients with type 2 diabetes mellitus (T2DM) before and after laparoscopic sleeve gastrectomy (LSG) and Roux-en-Y gastric bypass (RYGB). **(A)** Plasma level of C-peptide. **(B)** Plasma level of ghrelin. **(C)** Plasma level of GIP. **(D)** Plasma level of GLP-1. **(E)** Plasma level of insulin. **(F)** Plasma level of resistin. **(G)** Plasma level of leptin. **(H)** Plasma level of visfatin. **(I)** Plasma level of PAI-1. **(J)** Plasma level of glucagon. ^∗^*p* < 0.05; differences in significance level were determined using one-way ANOVA. 1 – healthy volunteers before breakfast; 2 – healthy volunteers after breakfast; 3 – obese patient without T2DM before breakfast; 4 – obese patient without T2DM after breakfast; 5 – obese patient with T2DM before breakfast; 6 – obese patient with T2DM after breakfast; 7 – 6 months after LSG before breakfast; 8 – 6 months after LSG after breakfast; 9 – 6 months after RYGB before breakfast; 10 – 6 months after RYGB after breakfast.

Disorders of carbohydrate and lipid metabolism can inhibit various compensatory mechanisms. A likely compensation mechanism could be an abnormal decrease in the total ghrelin level in obese patients without T2DM relative to healthy volunteers and obese patients with T2DM (Figure 2).

We analyzed patients after LSG surgery to determine the importance of lowering ghrelin levels in maintaining normal carbohydrate metabolism in obese patients. This surgery removes the fundus of the stomach (the main area of food addiction) that produces ghrelin. Basal ghrelin level was lower in patients operated on LSG than that in healthy donors and in patients before surgery. Thus, a low plasma total ghrelin level is a compensatory mechanism for IR in obese patients.

**FIGURE 3:**
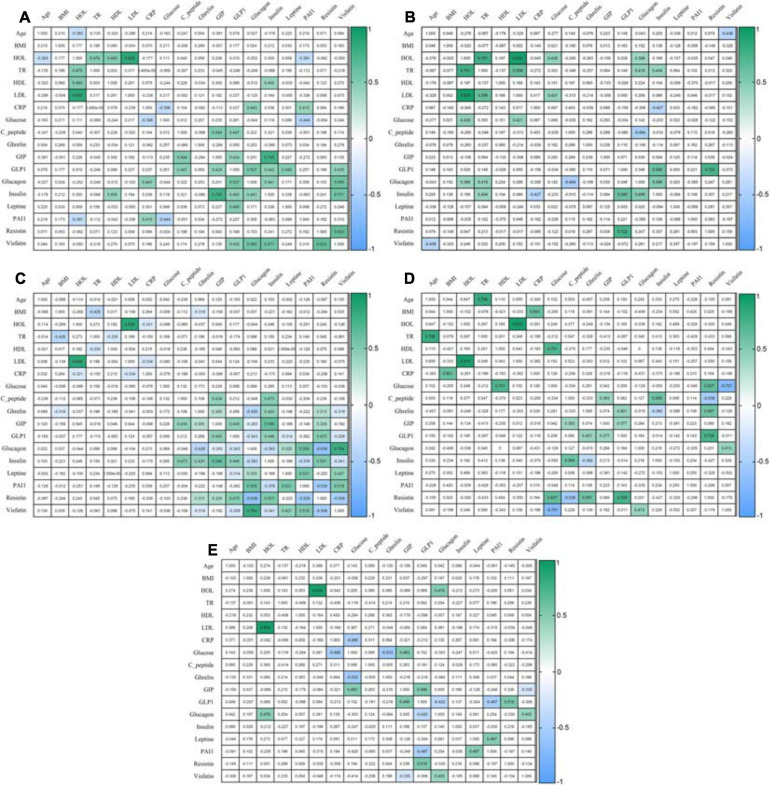
Correlation of mediators. **(A)** Correlation in the healthy volunteers; **(B)** Correlation in an obese patient without T2DM; **(C)** Correlation in an obese patient with T2DM; **(D)** Correlation in patients 6 months after LSG; **(E)** Correlation in patients 6 months after RYGB. The analysis was performed using Pearson’s test correlation.

**FIGURE 4:**
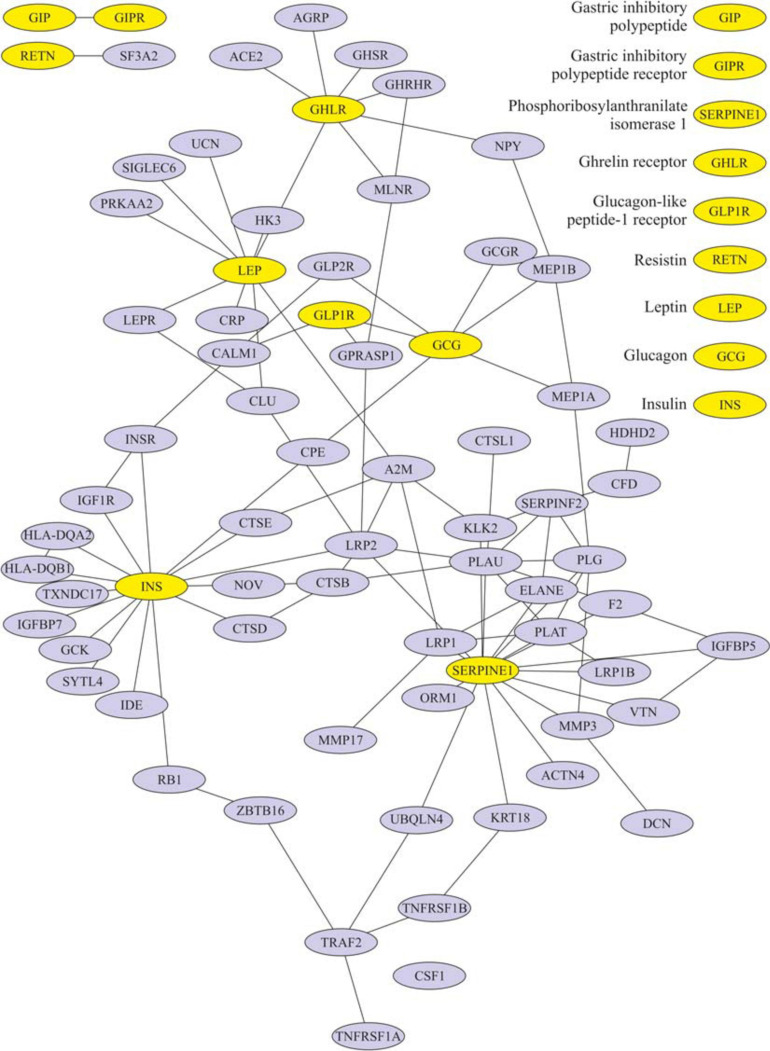
Subnetwork of the proteins insulin, ghrelin, glucagon-like peptide-1 (GLP-1), leptin, and their nearest neighbors. Light nodes represent the proteins under study; dark nodes are their nearest neighbors.

After eating, ghrelin levels increased in patients after LSG surgery. This may seem contradictory if we do not consider the correlation results and data from Gene Ontology and compare them in patients with T2DM before and after LSG surgery. The ghrelin level was positively correlated with insulin before surgery (*r* = 0.420) (Figure 3C) and negatively correlated in patients after LSG surgery (*r* = −392) (*p* < 0.05) (Figure 3D). We showed *in silico* that ghrelin was associated with insulin, leptin, glucagon, and CRP (Figure 4). In particular, we found *in silico* connections between ghrelin and insulin through several pathways: GHRL–MLNR–GPRASP1–LRP2–INS, GHRL–HK3–LEP–A2M–CTSE–INS, GHRL–HK3–LEP–CLU–CPE–INS, and GHRL–HK3–LEP–LEPR–CLU–CPE–INS (Figure 4). These pathways can be activated depending on the microenvironment. The high levels of insulin after breakfast and the associated mediators stimulated ghrelin production in an endocrine manner.

Ghrelin level negatively correlated with BMI and glucagon in patients with T2DM (Figure 3C) and with insulin after LSG (Figure 3D). Stimulation of ghrelin can occur not only by irritating the stomach’s fundus but also at the endocrine level.

### Ghrelin Is Associated With Incretins

Laparoscopic sleeve gastrectomy is an effective weight loss surgery with the removal of the central ghrelin production gastric zone. Ghrelin levels were negatively correlated with BMI and decreased in patients after surgery. Ghrelin levels were lower in patients after LSG than in patients after RYGB. The reason for the differences is the peculiarities of the surgery themselves. In patients after surgery, BMI decreased, and lipid metabolism indicators returned to normal but not glucose and CRP levels. Despite this, the patients, after LSG and RYGB, are characterized by low insulin levels.

The association of ghrelin and incretins confirms the revealed positive correlations between ghrelin and GIP in obese patients with T2DM (Figure 3C); in patients after LSG, positive correlations were noted between ghrelin and GLP-1 (Figure 3D). *In silico*, ghrelin has been shown to bind GLP-1 (Figure 4) via insulin or glucagon. In this regard, ghrelin is associated with GLP-1 and may affect GLP-1 levels after LSG in obese patients (*r* = 0.401) (Figure 3).

### Functional Annotation of Ghrelin and Its Receptors

The effects of ghrelin are multidirectional and depend on its isoforms and the activation of the corresponding receptors. There are several receptors for ghrelin. With the functional annotation of these receptor names, only one was found: G protein-coupled receptor (GPCR). This gene is designated FZD4 in the search results. The following functions were revealed in GO terms (Supplementary Table 4). The participation of FZD4 in the regulation of the morphogenesis of the organs of vision and the brain and the regulation of the secretion of steroid hormones, including progesterone, has been shown.

Enrichment analysis of the pathways for this receptor showed that GPCR (FZD4) belongs to the following signaling pathways:

R-HSA: 4641263 Regulation of FZD by ubiquitination;

R-HSA: 5099900 WNT5A-dependent internalizations of FZD4;

R-HSA: 5340588 RNF mutants show enhanced WNT signaling and proliferation.

Figure 4 shows that ghrelin is associated with the growth hormone secretagogue receptor (GHSR). The functional annotation of the GHSR gene is provided in the Supplementary Material (Supplementary Table 5). GHSR is associated with food responses and negative appetite regulation. In the gastrointestinal tract, the GHSR receptor is associated with food transit through the intestine and intestinal musculature contraction. GHSR is associated with the synthesis of neurotransmitters, norepinephrine, and catecholamines to synthesize growth hormones. GHSR negatively regulates the apoptosis of macrophages and myeloid cells and is associated with blocking inflammation: it inhibits tumor necrosis factor-alpha (TNF-a) and interleukin-6 (IL-6) functions.

Ghrelin is also associated with the growth hormone-releasing hormone receptor (GHRHR) (Figure 4). Functional annotation of GHRHR revealed the functions (signaling pathways were also not identified) and are listed in Supplementary Table 8. GHRHR was associated with the activation of the growth hormone-releasing receptor, regulating the production of growth hormone, and insulin-like growth factor. Additionally, receptor activation has been associated with the regulation of the sleep/wake cycle.

Thus, activation of GHSR has protective effects on carbohydrate and lipid metabolism.

### Functional Annotation of the Gastric Inhibitory Polypeptide Receptor and Glucagon-Like Peptide-1 Receptor Incretin Receptors

Incretins mediate their effects through the receptors GIPR and GLP-1R. According to our earlier data (Skuratovskaia, 2018), a special role in the disturbance of carbohydrate metabolism in obesity is played by the occurrence of polymorphisms in the GIPR and GLP-1R genes. To identify possible mechanisms of these genes’ participation in metabolic processes, functional annotation and analysis of their involvement in metabolic and signaling pathways were carried out (Supplementary Table 9).

Based on the functional annotation results, the GIPR gene was assigned to the following terms in the Gene Ontology category “biological process”: realization of mechanisms of development of many diseases, including those associated with digestion processes. GIPR has been associated with the development of a response to fatty acids, as well as with the processes responsible for the development of the pancreas from its formation to a mature structure, including islet cells that produce insulin, glucagon, and somatostatin.

Molecular functions corresponding to these biological processes were also identified: selective non-covalent interaction with a protein with hormonal activity (GO: 0017046, peptide hormone binding) and activity of the GIP receptor, that is, binding to GIP and signal transduction across the membrane to activate the G-protein (GO: 0016519, gastric inhibitory peptide receptor activity) (Usdin et al., 1993).

The functional annotation of GLP-1R was performed, which showed the participation of the GLP-1R gene in the regulation of adenylate cyclase activity (category “biological process”) (Supplementary Table 6).

In this regard, the effects of GLP-1R are not only implemented through glucose-dependent stimulation of insulin but are also associated with many other metabolic processes. Thus, GLP-1R activation can participate in the regulation of adenylate cyclase activity and cAMP-mediated signaling. Additionally, the relationship between GLP-1R and glucagon was shown, which was reflected in experimental studies: the level of glucagon positively correlated with GLP-1 in patients in the healthy donors (*r* = 0.527, *p* < 0.05) and negatively correlated with GLP-1 in patients with T2DM (*r* = −0.343, *p* < 0.05).

### Contribution of C-Peptide, Insulin, Leptin, PAI, Glucose-Dependent Insulinotropic Polypeptide, Glucagon-Like Peptide-1, Ghrelin, Resistin, and Visfatin to the Formation of Insulin Resistance

The data obtained indicate a change in carbohydrate and lipid metabolism, as the content of cholesterol, triglycerides, and LDL increased in both groups of patients (Supplementary Table 1).

The levels of hormones of the gastroduodenal zone, adipokines, and PAI-1 on an empty stomach (odd numbers of groups) and after a test breakfast (even numbers of groups) were studied in the following groups of patients: 1 and 2 – healthy volunteers, 3 and 4 – a comparison group of obese patients without T2DM, 5 and 6 – obese patients with T2DM, 7 and 8 – obese patients with T2DM 6 months after LSG, and 9 and 10 – obese patients with T2DM 6 months after RYGB (Figure 2). We measured mediator levels on an empty stomach and 60 min after the test breakfast, as the intensity of their production depends on the amount of nutrient intake.

Postprandial dynamics of mediators were observed only in patients with T2DM (groups 5 and 6). Significant differences between hormones (ghrelin, C-peptide, GIP, GLP-1, insulin, leptin, and resistin) and PAI-1 from other study groups were found in patients with T2DM. Thus, in patients with T2DM on an empty stomach and/or after a test breakfast, C-peptide, insulin, GIP, GLP-1, glucagon, ghrelin, leptin, PAI-1, and resistin were higher than those in obese patients without T2DM. The same changes in C-peptide levels, insulin leptin, PAI, and GIP were revealed; the maximum value was found in patients with T2DM after a test breakfast compared with the other groups.

Simultaneously, changes in the concentration of these mediators (C-peptide, insulin, GIP, GLP-1, glucagon, ghrelin, leptin, PAI-1, resistin, and visfatin) before and after the test breakfast did not depend on food intake. The GLP-1 and ghrelin levels reached maximum values in patients with T2DM relative to the other groups, but their change was associated with food intake.

In patients without T2DM, significant differences from the healthy volunteers were revealed only with respect to the ghrelin level, which was significantly lower. In general, in obese patients without T2DM, C-peptide, insulin, leptin, PAI, GIP, GLP-1, ghrelin, and resistin were significantly lower than those in obese patients with T2DM.

We investigated hormone and adipokine levels in patients with T2DM 6 months after LSG and RYGB bariatric surgeries.

In our study, all patients had hyperglycemia, normalization of lipid metabolism, and decreased body weight after 6 months.

Thus, in patients after LSG and RYGB surgeries, there was a decrease in C-peptide levels, insulin, leptin, PAI, GIP, GLP-1, ghrelin, resistin, and visfatin compared with obese patients with T2DM before surgery (Figure 2). After LSG, it was found that the level of ghrelin on an empty stomach and after breakfast was significantly lower than that in the other groups. The ghrelin level after the test breakfast was higher than the fasting values in LSG patients.

After RYGB, it was found that the level of GLP-1 did not change relative to the obese patients with T2DM before surgery, and the level of GIP after the test breakfast was lower compared with obese patients with T2DM (Figure 2). However, after RYGB, fasting, and the test breakfast, GLP-1 levels were higher than those in obese patients without T2DM (Figure 2).

The ghrelin level in patients after RYGB was higher than that in patients after LSG but lower than that in patients with T2DM before surgery (Figure 2). This finding indicates the regulatory role of these hormones on plasma glucose.

Visfatin levels changed only after RYGB. The concentration of visfatin in patients after RYGB was higher than obese patients without T2DM and compared with obese patients with T2DM (Figure 2).

Thus, C-peptide, insulin, leptin, PAI, GIP, GLP-1, ghrelin, resistin, and visfatin are closely related to carbohydrate and lipid metabolism and BMI. It contributes to the formation of IR in obese patients. However, the nature of these relationships is unclear.

#### Enrichment Assay for GIPR, GLP-1R, Insulin, Ghrelin, GIP, GLP-1, Leptin, and Resistin

Identifying the functions of the studied genes correlates with the data on participation in the corresponding processes of signal transduction in cells. The following pathways were identified in which the *GIPR* and *GLP-1R* genes are involved: the cAMP signaling pathway, GPCR ligand binding, G alpha(s) signaling events, and glucagon-type ligand receptors (Supplementary Table 9).

Thus, activation of GIPR and GLP-1R is associated with the following signaling pathways: cAMP, neuroactive interaction, secretin family receptor class B/2, glucagon-type ligand receptors, G alpha(s) signaling events, and GPCR ligand binding. It was shown that GIP was negatively correlated with glucagon in patients with T2DM (*r* = −0.283, *r* = −0.343, *p* < 0.05) (Figure 3C). In patients with T2DM after RYGB, glucagon levels negatively correlated with GLP-1 (*r* = −0.420, *p* < 0.05) (Figure 3E).

Interestingly, the GPCR receptor can also interact with ghrelin. In obese patients with T2DM, ghrelin positively correlated with GIP (*r* = 0.305), insulin (*r* = 0.420), and resistin (*r* = 0.313) and negatively correlated with BMI (*r* = −0.318), glucagon (*r* = −0.426), and visfatin (*r* = −0.319) (*p* < 0.05) (Figure 3C).

Pathway enrichment analysis for the remaining proteins under study was performed to assess the possible mechanisms of the involvement of key regulators of carbohydrate metabolism in metabolism and intracellular signaling processes. These were the identified pathways: FOXO-mediated transcription of oxidative stress, metabolism, and neuronal genes; synthesis, secretion, and deacylation of ghrelin.

The results obtained demonstrate the joint participation of the studied regulators of carbohydrate metabolism in incretin-mediated and ghrelin-mediated functions and metabolic processes. In this case, these factors’ mutual influence is implemented through a network of direct and indirect interactions.

## Discussion

C-peptide, ghrelin, GIP, GLP-1, insulin glucagon, PAI-1, resistin, leptin, and visfatin have been studied for assessing insulin sensitivity after bariatric surgery. It has been shown that these hormones have glucose-dependent secretion, have close relationships with each other, and have indicators of carbohydrate and lipid metabolism (Goktas et al., 2013; Pérez-Pevida et al., 2019). However, the results are multidirectional, and some relationships have not yet been deciphered. In our study, we combined an *in silico* and *in vivo* analysis unit.

Analyzing the data obtained, in obese patients with T2DM, the function of β-cells and the mechanism of substrate regulation works were not impaired, i.e., the higher the glucose level was, the more insulin the β-cells of the pancreas produced.

Interestingly, changes in the concentration of ghrelin were observed in various pathological conditions. Ghrelin is metabolically active with a negative metabolic balance (Al Qarni et al., 2017). At the excess intake of nutrients in obesity, the pathways that control energy balance became dysfunctional (Pöykkö et al., 2003; Al Qarni et al., 2017).

We showed *in silico* that ghrelin was associated with insulin, leptin, glucagon, and CRP. In particular, we found *in silico* connections between ghrelin and insulin through several pathways: GHRL–MLNR–GPRASP1–LRP2–INS, GHRL–HK3–LEP–A2M–CTSE–INS, GHRL–HK3–LEP–CLU–CPE–INS, and GHRL–HK3–LEP–LEPR–CLU–CPE–INS.

The level of ghrelin in obese patients with T2DM significantly increased relative to the other groups. The high levels of ghrelin may be due to the influence of high insulin levels in obese patients with T2DM, and its level depends on the level of other hormones.

The levels of C-peptide, insulin, GIP, GLP-1, leptin, and PAI-1 increased only in patients with T2DM. We have shown several links of ghrelin with hormones *in silico*: leptin, glucagon, insulin, GLP-1R, and PAI-1. Overall, this was consistent with our findings in obese patients.

The levels of total ghrelin and acyl-ghrelin are reduced in obese patients. The diacyl-ghrelin/acyl-ghrelin balance may change in obesity. The presence of one form or another can bind to different receptors and contribute to a change in ghrelin’s effects. Interestingly, ghrelin levels were lower in obese patients without T2DM than in obese patients with T2DM and healthy volunteers. We emphasize this as a compensatory mechanism for maintaining normal carbohydrate and lipid metabolism in obesity. In patients after LSG and RYGB, against the background of a decrease in BMI, the blood plasma content of ghrelin was less than that in obese patients with T2DM before surgery.

Other authors have shown that ghrelin deficiency does not prevent diet-induced obesity (McFarlane et al., 2014). This proves that all patients showed hyperglycemia after 6 months, in contrast to our previous results (Skuratovskaia et al., 2019), where normoglycemia was noted after 12 months. Patients 6 months after LSG and RYGB are in an adaptive rehabilitation period, as evidenced by high glucose and C-reactive protein levels, indicating an acute phase of inflammation.

We believe that the compensation mechanism consists of the activation of specific receptors.

To elucidate the interaction pathways, we performed an *in silico* analysis of ghrelin receptors. It is known that preproghrelin undergoes posttranslational processing to form at least five products. Acyl-ghrelin is biologically active and interacts with the GHSR, and deacyl-ghrelin acts independently of GHSR1 (Gray et al., 2019). Acyl-ghrelin stimulates food intake, gastrointestinal motility, lipogenesis, and glycemia and reduces energy expenditure and insulin secretion/sensitivity (Gray et al., 2019). Deacyl-ghrelin inhibits food intake, gastrointestinal motility, and glycemia and stimulates insulin secretion through an as yet unknown receptor (Cui et al., 2017). Deacyl-ghrelin prevents the development of obesity and positively affects insulin sensitivity (Cui et al., 2017).

According to the latest data, the ghrelin receptors are GPCRs, GHSR1a, GHSR1b, ghrelin receptor-like receptor, and seven- and five-transmembrane GPCR to motilin (Sanger and Furness, 2016).

According to the functional annotation of ghrelin receptors, it is known that it activates three receptors: GPCR, GHSR, and GHRHR (Sanger and Furness, 2016). GPCRs have been associated with FZD4 modifications and the proliferative Wnt signaling pathway. GHSR has been associated with food responses, gut muscle contraction, and neurotransmitter and growth hormone synthesis. The role of GHSR in the immune response is interesting. GHSR blocks TNF-a and IL-6 functions and apoptosis of macrophages and myeloid cells (Cabral et al., 2017). GHRHR is associated with the production of growth factors, somatotropin, and insulin-like growth factor. Additionally, receptor activation is associated with the regulation of the sleep/wake cycle (Sanger and Furness, 2016).

It has been shown that ghrelin stimulates appetite by activating the hypothalamus and activates lipogenesis, leading to obesity (Qader et al., 2005). However, in our study, there was no relationship with lipid metabolism indicators.

It has been suggested that ghrelin and its receptor expressed in α-cells of the pancreas affect glucose metabolism not only by directly inhibiting the stimulation of insulin secretion by glucose (Qader et al., 2005) but also by stimulating glucagon secretion by α-cells (Chuang et al., 2011). In patients with T2DM, ghrelin positively correlated with insulin (*r* = 0.402, *p* < 0.05) and negatively correlated with glucagon (*r* = −0.426, *p* < 0.05), which indicates a violation of the relationship between hormones.

Thus, the predominance of one form or another of ghrelin and the activation of its receptors determine the fate of many links in the pathogenesis of IR in obesity. Our study measured total ghrelin; however, all its posttranslational modifications and effector pathways are of interest.

### Association of Ghrelin With Incretins

In healthy people, the effects of ghrelin and GLP-1 on glucose metabolism are oppositely directed, but ghrelin can modulate postprandial GLP-1 secretion (Gagnon et al., 2015; Tong et al., 2016, 1; Gray et al., 2019) *in silico*. It was found that ghrelin interacts with GLP-1 and glucagon: GHRL–HK3–LEP–CRP–GLP2R–GCG, GHRL–HK3–LEP–LEPR–CLU–CPE–GCG, and GHRL–NPY–MEP1B–GCG. The incretin receptors GIPR and GLP-1R have been implicated in the regulation of the cAMP signaling pathway.

We obtained earlier data on the important role of defects in incretin receptors in IR development. Thus, it was shown that the genotypes associated with an increased risk of developing T2DM, CC *rs1042044*, and AA *rs6923761* of the *GLP-1R* gene polymorphism, are characterized by an increase in the plasma level of incretin in the group of obese patients with T2DM and serum glucose levels in the group of obese patients without T2DM (Skuratovskaia, 2018). Impaired activation of GIPR and GLP-1R contributes to a decrease in the secretion of insulin and ghrelin and, on the contrary, to an increase in leptin and glucagon in the circulation, which contributes to the formation of insulin resistance in obese patients with T2DM.

Glucagon-like peptide-1 receptor is involved in the glucagon-type ligand-receptor family signaling pathway, which regulates the activity of GPCRs from the class II/B secretin receptor subfamily (Yabe and Seino, 2011). GLP-1R is synthesized in intestinal L cells in response to the presence of glucose and fatty acids (Tong et al., 2016). Most GLP-1 is in the GLP-1 (7–36) amidated form; some are the GLP-1–GLP-1 (7–37) form (Farr et al., 2016). GLP-1 circulates to the pancreas, where it binds to GLP-1R (Tong et al., 2016). GLP-1R is a transmembrane protein and a member of the B family of GPCRs with an N-terminal extracellular domain (Gagnon et al., 2015).

We performed functional annotation and analysis of the involvement of the *GLP-1R* gene in the signaling pathways, indicating its participation in the initiation of cAMP signaling, neuroactive interaction, and the provision of cell response to a stimulus in the form of glucagon, which was reflected in experimental studies: in patients of the healthy volunteers, the glucagon level positively correlated with GLP-1 (*r* = 0.527, *p* < 0.05). In obese patients with and without T2DM, as well as in healthy volunteers, GLP-1 and insulin levels were positively correlated. However, after RYGB surgery, no such relationship was found. GLP-1 and glucagon levels were negatively correlated.

Although GLP-1 and glucagon are formed from a common precursor (Hayashi, 2019), glucagon levels did not change among the study groups.

We have shown the dependence of the total ghrelin content on other mediators, such as insulin, leptin, GIP, GLP-1, and PAI-1. Our data suggest the cooperative interaction of mediators associated with ghrelin, which realizes their obesity effects, depending on the presence or absence of IR. However, the effects of ghrelin may depend on how its effects are realized.

We used models of different surgeries to study in detail the mutual regulation of hormones. LSG removes most of the ghrelin-producing zone in the stomach. Unusually, the ghrelin level after the breakfast test did not decrease but was significantly higher than the fasting values in patients after LSG. This fact may indicate that other additional stimulatory signals exist for the secretion of ghrelin, acting differently from the mechanical stimulation of the cells of the fundus of the stomach and depending on remote regulation by the intake of nutrients. Moreover, it is believed that the return of insulin sensitivity is facilitated by a decrease in the inhibitory effect of ghrelin on insulin (McFarlane et al., 2014), which is consistent with our results on the construction of a network of interactions of the studied proteins. We found an indirect interaction of ghrelin and insulin (through three nodes: MLNR, GPRASP1, and LRP2). Of interest is the study of some links of this pathway: MLNR is a motilin receptor involved in hormone binding, GPRASP1 promotes the degradation of G protein-coupled receptors in lysosomes (Whistler et al., 2002), and LRP2 is required for insulin-dependent internalization of IR (Seo et al., 2020).

According to the results of the study, two groups of pathways were identified in which the studied molecules are involved: FOXO-mediated transcription of genes for oxidative stress, metabolism, and neurons (through the participation of *INS* and *RETN*) and the synthesis, secretion, and diacylation of ghrelin (with the involvement of *GCG*, *GHRL*, *INS*, *LEP*, and *GIP*). The results obtained demonstrate the joint participation of the studied regulators of carbohydrate metabolism in incretin-mediated and ghrelin-mediated functions and metabolic processes. Simultaneously, the mutual influence of these factors is realized through a network of direct and mediated interactions.

Interestingly, the GPCR can also interact with ghrelin. Moreover, this same receptor can be activated by GLP-1 and GIP (Abdullah et al., 2016). In obese patients with T2DM, ghrelin positively correlated with GIP (*r* = 0.305), insulin (*r* = 0.420), and resistin (*r* = 0.313) and negatively correlated with BMI (*r* = −0.318), glucagon (*r* = −0.426), and visfatin (*r* = −0.319) (*p* < 0.05). Thus, C-peptide, insulin, leptin, PAI, GIP, GLP-1, ghrelin, resistin, and visfatin are closely associated with carbohydrate and lipid metabolism and BMI in obese patients, which contributes to the formation of IR in obesity.

The use of ghrelin receptor antagonists is effective in correcting carbohydrate metabolism. There is one clinical trial in humans that promoted glucose-dependent insulin secretion by blocking the GHSR1a receptor (Schalla and Stengel, 2019). Despite the significant effect, ghrelin did not have the desired effect in treating metabolic disorders, and the drug is currently used to treat insomnia (Schalla and Stengel, 2019).

The animal models have recently shown that GHS-R1b antagonists lead to decreased food intake (Schalla and Stengel, 2019). The development of GHSR antagonists leads to unpredictable results and requires a detailed study of the mechanisms of action. The predominance of one form or another of ghrelin and the activation of its receptors determine the fate of many links in the pathogenesis of IR in obesity. The search for new receptors for ghrelin and the study of posttranslational modifications of ghrelin will make it possible to study the regulation of metabolic disorders more closely. Further studies can help identify the mechanisms of participation of ghrelin in metabolic disorders in patients.

## Conclusion

A decreased level of total ghrelin before and after breakfast is typical only for obese patients without insulin resistance. Its increased level is typical for obese patients with T2DM. Ghrelin exerts its effects through the receptors GPCR, GHSR, and GHRHR, which are associated with proliferative, inflammatory, and neurohumoral signaling pathways and regulate responses to nutrient intake. The signaling pathway for realizing the effects of ghrelin, GIP, GLP-1, and GCG lies through the class of GPCRs. This demonstrates common regulatory mechanisms and cross-talk between ghrelin and incretins. This will help draw attention to posttranslational modifications and the associated ghrelin and incretin receptors for use in targeted therapy of insulin resistance.

The results obtained demonstrate the joint participation of the studied regulators of carbohydrate metabolism in incretin-mediated and ghrelin-mediated functions and metabolic processes. Simultaneously, the mutual influence of these factors is realized through a network of direct and mediated interactions. A cooperative exchange of ghrelin and mediators associated with it, such as insulin, leptin, GIP, GLP-1, glucagon, and PAI-1, was revealed, which realizes their effects on obesity. Insulin, leptin, GIP, GLP-1, glucagon, and PAI-1 are linked directly or indirectly (see the “Results” section for more details). Thus, ghrelin and incretins can modulate insulin, leptin, glucagon, and PAI-1.

This does not exclude the influence of insulin and glucose on the joint change in mediators due to other factors not included in the study. The influence of the microenvironment on the production of these mediators should be taken into account.

## Data Availability Statement

The raw data supporting the conclusions of this article will be made available by the authors, without undue reservation.

## Ethics Statement

The studies involving human participants were reviewed and approved by all the study participants provided informed consent to participate in a research study. The study was carried out in accordance with the World Medical Association (WMA) Declaration of Helsinki (2000) and the Protocol to the Convention on Human Rights and Biomedicine (1999). The Local Ethical Committee approved the study protocol of the Innovation Park of the Immanuel Kant Baltic Federal University (Protocol No. 4 from October 23, 2013). The patients/participants provided their written informed consent to participate in this study.

## Author Contributions

DS and MV conceptualized the study. LL and EK performed the validation. AK did the formal analysis. ES and AK wrote and prepared the original draft and performed the visualization. DS, MV, and LL reviewed and edited the article. NC did the bioinformatics analysis. AK and DS performed the experimental work. All authors contributed to the article and approved the submitted version.

## Conflict of Interest

The authors declare that the research was conducted in the absence of any commercial or financial relationships that could be construed as a potential conflict of interest.
