# Supplementary material for: The Links of Ghrelin to Incretins, Insulin, Glucagon, and Leptin After Bariatric Surgery
Source: Front Genet. 2021 Apr 20;12:612501. doi: 10.3389/fgene.2021.612501 (PMC8093791; doi:10.3389/fgene.2021.612501)
Supplement: Supplementary file 1 [file Data_Sheet_1.docx]

Supplementary Material

|  | **Control group** | **Obese patient without T2D** | **Obese patient with T2D** | **6 months after LSG** | **6 months after RYGB** |
| --- | --- | --- | --- | --- | --- |
|  | 1 | 2 | 3 | 4 | 5 |
| **Cholesterol**  **(< 5.2)**  **mmol/l** | 4.51 (4.21-5.02) | 5.32 (4.41-6.04)  р 1-2 * | 5.2  (4.33-6.01) | 4.19 (3.63-5.09)  р 2-4 **  р 3-4 ** | 4.46 (4.09-5.07)  р 2-5 **  р 3-5 * |
| **Triglycerides**  **(< 2.53)**  **mmol/l** | 1.11 (0.75-1.69) | 1.9 (1.25-2.41)  р 1-2 *** | 1.86 (1.41-2.44)  р 1-3 *** | 1.26  (0.76-1.4)  р 2-4 **  р 3-4 *** | 1.16   (1-1.37)  р 2-5 ***  р 3-5 *** |
| **High-density lipoproteins (HDL)**  **(0.78 – 1.81) mmol / l** | 1.19 (1.02-1.38) | 1.23 (0.98-1.45) | 1.09 (0.89-1.24) | 1.05 (0.98-1.29) | 1.04 (0.95-1.18) |
| **Low-density lipoproteins (LDL)**  **(0.00 – 3.4) mmol / l** | 2.67 (2.3-3.04) | 3.11 (2.67-3.62)  р 1-2 * | 2.99 (2.46-3.55)  р 1-3 * | 2.56 (1.93-2.81)  р 2-4 **  р 3-4 * | 2.6  (2.28-3.04)  р 2-5 *  р 3-5 * |
| **C-reactive protein**  **(>6.0)**  **mg/l** | 1.09 (0.89-1.93) | 9.23 (5.04-15.11)  р 1-2 *** | 4.75 (1.87-9.22)  р 1-3 ***  р 2-3 ** | 13.86 (9.24-19.29)  р 1-4 ***  р 3-4 ** | 7.66  (4.99-12)  р 1-5 ***  р 4-5 * |
| **Glucose before breakfast**  **(3.9-6.4)**  **mmol/l** | 5.00  (4.65-5.42) | 5.55 (5.03-6.24)  р 1-2 ** | 6.28 (5.64-7.04)  р 1-3 ***  р 2-3 ** | 6.15 (5.17-9.02)  р 1-4 *** | 8.46 (6.86-10.74)  р 1-5 ***  р 2-5 ***  р 3-5 ** |
| **Glucose after breakfast**  **mmol/l** | 5.54 (4.76-5.77) | 6.4  (5.55-7.75)  #  р 1-2 * | 8.05 (6.88-9.88)  #  р 1-3 ***  р 2-3 ** | 7.99 (7.86-8.12) | 7.12 (6.16-9.78)  р 1-5 * |

**Supplementary Table 1.** The biochemical parameters of the lipid and protein metabolism. liver enzymes and glucose in the obese patient's serum with and without T2DM.

Note: *– p<0.05, ** – p<0.001, *** – p<0.0001; # - differences in glucose before and after breakfast; differences in significance level were determined using the Mann-Whitney criterion for two independent samples (Me(Q1–Q3)). 1- Control group; 2- Obese patient without T2D; 3- Obese patient with T2D; 4- 6 months after LSG; 5- 6 months after RYGB.

| Biochemical parameters | Sex | Control group  n=42 | Obese patient without T2DM  n=90 | Obese patient with T2DM  n=72 | RYGB  n=31 | LCG  n=23 |
| --- | --- | --- | --- | --- | --- | --- |
|  |  | 1 | 2 | 3 | 4 | 5 |
| Cholesterol | Male  (< 5,2) mmol/l | n=17  4,5 (4,31-5,5) | n=28  4,14 (3,42-4,83) | n=20  5,12 (4,31-5,97) | n=7  4,44 (4,14-5,31) | n=5  4,96 (4,08-5,08) |
|  | Female  (< 5,2) mmol/l | n=25  4,98 (4,39-5,2)  p=0,2 | n=62  4,9 (4,21-5,79)  p=0,0044 ** | n=52  5,29 (4,35-5,98)  p=0,97 | n=24  4,46 (4-5,18)  p=1,0 | n=18  4,66 (3,73-5,28)  p=0,82 |
| Triglycerides | Male  (< 2,53) mmol/l | n=17  0,71 (0,56-1,23) | n=28  1,26 (0,89-2,34) | n=20  1,93 (1,42-2,9) | n=7  1,29 (0,82-1,52) | n=5  1,39 (1,13-1,78) |
|  | Female  (< 2,53) mmol/l | n=25  0,67 (0,53-0,89)  p=0,2 | n=62  1,46 (1-1,93)  p=0,71 | n=52  1,85 (1,37-2,32)  p=0,42 | n=24  1,14 (1-1,36)  p=0,42 | n=18  1,23 (0,86-1,59)  p=0,43 |
| High-density lipoproteins (HDL) | Male  (0,78 – 1,81) mmol/l | n=16  1,23 (1,05-1,41) | n=28  0,95 (0,81-1,07) | n=20  0,99 (0,82-1,14) | n=7  0,95 (0,89-1,11) | n=5  1,29 (1,02-1,34) |
|  | Female  (0,78 – 2,2) mmol/l | n=25  1,77 (1,54-2,19)  p˂0,0001 *** | n=62  1,25 (1,04-1,62)  p˂0,0001 *** | n=52  1,13 (0,97-1,33)  p=0,03 * | n=24  1,19 (1,01-1,35)  p=0,06 | n=18  1,13 (0,98-1,3)  p=0,53 |
| Low-density lipoproteins (LDL) | Male  (˂ 3,4) mmol/l | n=16  2,67 (2,44-2,92) | n=28  2,41 (1,84-2,97) | n=20  3,08 (2,57-3,39) | n=7  2,65 (2,28-3,35) | n=5  2,66 (2,44-3,02) |
|  | Female  (˂ 3,4) mmol/l | n=25  2,77 (2,34-3,01)  p=0,65 | n=62  2,9 (2,38-3,43)  p=0,01 ** | n=52  2,99 (2,48-3,54)  p=0,86 | n=24  2,62 (2,32-2,9)  p=0,64 | n=18  2,79 (2,07-3,12)  p=0,97 |
| C-reactive protein (CRP) | Male  (>6.0) mg/l | n=14  1,02 (0,47-2,21) | n=17  6,92 (4,11-12,03) | n=18  5,78 (3,55-9,2) | n=7  1,21 (0,7-8,57) | n=5  4,87 (2,62-6,94) |
|  | Female  (>6.0) mg/l | n=23  1,08 (0,58-1,89)  p=1 | n=41  6,57 (2,67-9,02)  p=0,19 | n=46  7,16 (2,22-15,51)  p=0,39 | n=24  2,83 (1,5-4,67)  p=0,59 | n=18  3,54 (1,75-5,18)  p=0,97 |
| Glucose before breakfast | Male  (3,9-6,4) mmol/l | n=17  5,26 (4,8-5,44) | n=28  5,68 (5,05-6,48) | n=20  9,3 (7,23-10,52) | n=7  5,43 (4,62-6,48) | n=5  4,47 (3,94-5,21) |
|  | Female  (3,9-6,4) mmol/l | n=25  5,13 (5,03-5,28)  p=0,99 | n=62  5,35 (4,85-6,09)  p=0,42 | n=52  6,9 (5,57-8,76)  p=0,01 ** | n=24  4,86 (4,21-5,64)  p=0,38 | n=18  4,73 (4,13-5,48)  p=0,48 |
| Glucose after breakfast | Male | n=4  4,58 (4,11-5,18) | n=31  8,04 (6,84-8,66) | n=9  10,37 (7,96-12,72) | n=7  6,92 (3,86-7,72) | n=5  5,81 (5,43-6,43) |
|  | Female | n=14  5,5 (4,27-6,48)  p=0,26 | n=21  6,43 (5,6-8,66)  p=0,002 ** | n=31  8,04 (6,84-8,66)  p=0,04 * | n=20  5,93 (4,69-6,6)  p=0,93 | n=14  5,36 (4,77-7,25)  p=0,67 |

**Supplementary Table 2.** Sex differences of biochemical parameters in the serum of obese patients with/without T2DM.

Note: *– p<0.05, ** – p<0.001, *** – p<0.000; differences in significance level were determined using the Mann-Whitney criterion for two independent samples (Me(Q1–Q3)). 1- Control group; 2- Obese patient without T2DM; 3- Obese patient with T2DM; 4- 6 months after LSG; 5- 6 months after RYGB.

| Indicators  ng/ml  Study groups | | Sex | С–peptide | Grelin | GIP | GLP–1 | Glucagon | Insulin | Leptine | PAI–1 | Resistin | Visfatin |
| --- | --- | --- | --- | --- | --- | --- | --- | --- | --- | --- | --- | --- |
| Control group n=14 | 1 | male | n=2  292,7  (227,8-357,6) | n=4  96,73  (64,56-119,8) | n=4  42,16  (29,02-50,40) | n=2  9,95  (7,61-12,29) | n=5  227,8  (292,7-357,6) | n=4  46,91  (28,97-96,03) | n=2  145,5 (117,6-173,5) | n=4  991,4 (904,3-1109) | n=4  2135  (537,1-2627) | n=2  1067  (938,6-1195) |
|  |  | female | n=9  306,5 (235,8-346,2)  p=0,90 | n=9  79,45  (48,39-93,90)  p=0,41 | n=9  33  (23,87-43,54)  p=0, 41 | n=9  13,65  (10,81-16,17)  p=0,21 | n=9  306,5 (235,8-346,2)  p=0,69 | n=9  43,82  (34,75-87,67)  p=0,93 | n=9  427,1  (246,1-555,3)  p=0,07 | n=9  1146  (892,3-1581)  p=0,33 | n=9  1362  (571,8-2581)  p=0,71 | n=9  998,5 (514,4-1178)  p=0,72 |
|  | 2 | male | n=2  483,8  (420,7-546,9) | n=5  58,08  (50,2-77,54) | n=5  107,4  (74,64-135,6) | n=4  13,50  (9,35-15,36) | n=5  483,8  ( 420,7-546,9) | n=5  99,31  (52,08-130,0) | n=5  330,1  (180,4-501,2) | n=5  935,2  (759,6-1030) | n=5  1053(598,3-1707) | n=3  608,8  (445,8-744,1) |
|  |  | female | n=8  422,0  (356,0-746,8) | n=10  61,67  (51,14-92,09)  p=0,61 | n=10  112,1  ( 63,04-164,5)  p=0,61 | n=10  14,87  (9,563-)  p=0,47 | n=10  422,0  (356,0-746,8)  p=0,13 | n=10  119,6  (49,67-207,6)  p=0,45 | n=10  399,0 (167,3-582,7)  p=0,61 | n=10  803,5  (690,2-944,8)  p=0,38 | n=10  959,3  (505,5-3979)  p=0,70 | n=10  636,5  (355,0-1237)  p=0,98 |
| Obese patient without T2D n=22 | 1 | male | n=4  442,9  (430,7-452,9) | n=4  95  (54,07-99,18) | n=4  50,18  (33,45-53,91) | n=4  19,73  (8,25-21,97) | n=4  442,9  (430,7-450,9) | n=4  42,14  (39,50-90,13) | n=4  23,67  (15,73-567,3) | n=4  1330  (1296-1415) | n=4  1639  (1548-1749) | n=4  2463  (969,0-2631) |
|  |  | female | n=15  584,6  (541,5-754,5)  p=0,006 | n=15  58,2  (41,09-92,61) p=0,40 | n=15  42,0  (26,81-79,85)  p=0,73 | n=15  6,67  (4,54-16,89)  p=0,17 | n=15  584,6  ( 541,5-754,5)  p=0,22 | n=15  140,8  (57,97-185,0)  p=0,02 | n=15  1623  (1259-1759)  p=0,001 | n=15  1581  (1423-2152)  p=0,02 | n=14  1545  (1248-1811)  p=0,79 | n=15  569,0  (390,2-2326)  p=0,06 |
|  | 2 | male | n=4  1063  (980,5-1093) | n=4  101,0  (61,36-106,3) | n=4  120,5  (118,3-273,7) | n=4  31,41  (15,09-36,45) | n=4  1063  (980,5-1093) | n=4  151,4  (128,4-280,7) | n=4  18,34  (14,23-1088) | n=4  1152  (1036-1784) | n=4  1264  (1193-1900) | n=4  2200  (861,5-2705) |
|  |  | female | n=15  778,4  (527,2-940,6)  p=0,08 | n=15  54,93  (12,73-93,3)  p=0,22 | n=15  60,34  ( 20,74-148,5)  p=0,08 | n=14  6,4  (2,37-16,36)  p=0,01 | n=14  778,4  ( 527,2-940,6)  p=0,04 | n=14  203,7  (99,9-228,8)  p=0,95 | n=15  1428  (978,4-1493)  p=0,08 | n=15  1586  (1365-2008)  p=0,26 | n=13  1314  (522,0-1373)  p=0,62 | n=14  503,6  (323,6-2056)  p=0,13 |
| Obese patient with T2D  n=54 | 1 | male | n=11  476  (251,2-525,5) | n=10  64,65  (56,79-112,8) | n=10  69,47  ( 22,5-134,1) | n=10  11,74  (8,11-43,70) | n=10  476,0  ( 251,2-525,5) | n=12  182,7  (29,99-779,8) | n=11  1064  (287,2-6437) | n=8  1811  (1030-10499) | n=10  1086  (378,0-6522) | n=10  1091  (800,1-1184) |
|  |  | female | n=17  466,6  (346,4-2404)  p=0,25 | n=20  97,51  (73,91-135,9)  p=0,11 | n=19  79,26  (56,38-175,3)  p=0,20 | n=16  43,44  ( 24,58-64,75)  p=0,05 | n=20  466,6  (346,4-2404)  p=0,11 | n=20  339,4  (145,3-643,6)  p=0,13 | n=19  6303  (2135-8489)  p=0,01 | n=15  7695  (1725-18079)  p=0,05 | n=16  3325  (648,2-6936)  p=0,38 | n=18  890,6  (112,80-1287)  p=0,75 |
|  | 2 | male | n=9  490  (405,1-755 | n=13  94,62  (58,78-112,3) | n=11  174,3  (64,8-357,4 | n=11  20,88  (18,85-27,47) | n=11  490,2  ( 405,1-755,0) | n=11  553,4( 78,63-2189) | n=11  1730  (633,8-3360) | n=11  1664  (1592-10024) | n=9  1737  (1039-5123) | n=11  1470  (1181-2233) |
|  |  | female | n=18  519,6  (435,3-4135)  p=0,55 | n=22  98,57  (76,71-138,3)  p=0,28 | n=20  310  (215,7-646,9)  p=0,09 | n=20  41,46  (15,31-67,42)  p=0,27 | n=21  519,6  (435,3-4135)  p=0,80 | n=20  474,7( 276,1-1708) p=0,63 | n=19  5363  (2996-10004)  p=0,002 | n=20  10054 (2564-15475)  p=0,03 | n=16  4214  (1444-8009)  p=0,26 | n=21  961,6  (320,6-1464)  p=0,16 |
| RYGB | 1 | male | n=10  438,4  (211,9-  438,4) | n=9  61,33  (17,74-105,0) | n=9  60,25  (21,55-111,8) | n=5  31,65  (4,66-63,50) | n=10  303,0  (146,9-559,0) | n=7  95,84  (48,10-116,0) | n=9  648,0  (211,0-1667) | n=10  2080  (1099-7882) | n=6  3109  (1481-7295) | n=8  827,9  (116,0-1890) |
|  |  | female | n=17  410,3  (269,3-877,6)  p=0,60 | n=14  74,0  (58,77-86,16)  p=0,78 | n=14  64,34  (41,73-107,0)  p=0,78 | n=13  9,91  (6,56-41,19)  p=0,71 | n=17  486,1  (208,5-666,8)  p=0,28 | n=14  107,2  (48,21-214,6)  p=0,58 | n=14  1056  (811,0-3059)  p=0,10 | n=15  3571  (1996-7181)  p=0,28 | n=9  2031  (783,1-6561)  p=0,60 | n=17  1001  (418,8-1803)  p=0,62 |
|  | 2 | male | n=10  562,0  (291,1-907,7) | n=8  50,86  (16,81-81,20) | n=8  133  (70,83-205,2) | n=8  29,26  (13,98-44,43) | n=8  431,9  (204,3-9956) | n=8  152,3  (70,37-377,8) | n=8  387,0  (53,26-1286) | n=8  3627  (1661-3627) | n=5  1667  (1118-8237) | n=10  1244  (233,3-5425) |
|  |  | female | n=14  509,0  (362,7-939,6)  p=0,93 | n=13  64,53  (37,53-78,85)  p=0,37 | n=12  161,2  (98,7-236,1)  p=0,34 | n=12  32,69  (15,78-63,25)  p=0,52 | n=16  462,4  (266,2-780,2)  p=0,65 | n=12  133,7  (69,36-305,6)  p=0,92 | n=13  805,0  (528,6-2649)  p=0,08 | n=14  3248  (2128-9732)  p=0,92 | n=10  2639  (356,3-5160)  p=0,85 | n=16  797,8  (390,5-1639)  p=0,69 |
| LCS | 1 | male | n=4  769,0  (442,1-4801) | n=3  26,33  (14,71-96,0) | n=3  295,5(26,51-456,0) | n=2  41,30(8,59-74,0) | n=4  312,9(111,7-10001) | n=3  111,9  (96,74-438,5) | n=3  1599  (724,5-2859) | n=3  4759  (1462-7569) | n=2  3349  (130,4-6567) | n=4  570  (39,36-13326) |
|  |  | female | n=22  461,5  (216,4-838,7)  p=0,24 | n=20  19,57  (15,19-94,07)  p=0,95 | n=20  50,96(16,53-107,9)  p=0,22 | n=16  16,21(3,09-40,0)  p=0,45 | n=22  269,9  (171,1-465,9)  p=0,64 | n=20  75,04  (34,64-200,6)  p=0,34 | n=21  1346  (365,6-3365)  p=0,78 | n=18  1985  (1020-8998)  p=0,59 | n=18  2081  (555,9-3448)  p=0,84 | n=20  p=609,5  (298,6-1137)  p=0,66 |
|  | 2 | male | n=5  735,6  (296,6-4350) | n=4  45,8(17,11-80,55) | n=3  54,(102,6-519,4) | n=4  29,(11,36-67,42) | n=5  459,(147,3-6759) | n=4  143,3  (55,70-534,3) | n=4  1141  (797,2-1861) | n=4  5478  ( 1782-18826) | n=2  5340  (3958-6723) | n=5  1166  (423,5-2746) |
|  |  | female | n=20  641,9  (339,1-1273)  p=0,96 | n=19  36,31  (15,67-84,31)  p=0,95 | n=18  165,4(103,4-254,1)  p=0,42 | n=17  20,81(5,79-53,43)  p=0,62 | n=21  278,2  (240,0-557,5)  p=0,74 | n=18  167,1  (73,14-280,0)  p=0,88 | n=19  1148  (255,3-2860)  p=0,95 | n=18  1761  (856,6-5979)  p=0,21 | n=12  1765  (1053-5572)  p=0,34 | n=20  721,4  ( 378,0-970,3)  p=0,39 |

**Supplementary Table 3.** Sex differences of mediators in plasma in obese patients with and without type 2 diabetes before and after the test breakfast

Note: *– p<0.05, ** – p<0.001, *** – p<0.000; differences in significance level were determined using the Mann-Whitney criterion for two independent samples (Me(Q1–Q3)). 1- Control group; 2- Obese patient without T2DM; 3- Obese patient with T2DM; 4- 6 months after LSG; 5- 6 months after RYGB.

| **GeneOntology** | **Biological process** |
| --- | --- |
| GO:0150012 | positive regulation of neuron projection arborization |
| GO:0110135 | Norrin signaling pathway |
| GO:0001553 | luteinization |
| GO:0035929 | steroid hormone secretion |
| GO:0042701 | progesterone secretion |
| GO:0042813 | Wnt-activated receptor activity |
| GO:0061300 | cerebellum vasculature development |
| GO:0061298 | retina vasculature development in camera-type eye |
| GO:0061299 | retina vasculature morphogenesis in camera-type eye |
| GO:0061301 | cerebellum vasculature morphogenesis |
| GO:0061304 | retinal blood vessel morphogenesis |
| GO:0150011 | regulation of neuron projection arborization |

**Supplementary Table 4.** Functional annotation of GHCR (FZD4).

| **GeneOntology** | **Biological process** |
| --- | --- |
| GO:0042226 | interleukin-6 biosynthetic process |
| GO:0120058 | positive regulation of small intestinal transit |
| GO:0032095 | regulation of response to food |
| GO:0001616 | growth hormone secretagogue receptor activity |
| GO:0014061 | regulation of norepinephrine secretion |
| GO:0016520 | growth hormone-releasing hormone receptor activity |
| GO:0032096 | negative regulation of response to food |
| GO:0032097 | positive regulation of response to food |
| GO:0032108 | negative regulation of response to nutrient levels |
| GO:0032109 | positive regulation of response to nutrient levels |
| GO:0048243 | norepinephrine secretion |
| GO:1902683 | regulation of receptor localization to synapse |
| GO:0010700 | negative regulation of norepinephrine secretion |
| GO:0032099 | negative regulation of appetite |
| GO:0032100 | positive regulation of appetite |
| GO:0033604 | negative regulation of catecholamine secretion |
| GO:0043134 | regulation of hindgut contraction |
| GO:0045408 | regulation of interleukin-6 biosynthetic process |
| GO:1905562 | regulation of vascular endothelial cell proliferation |
| GO:0099645 | neurotransmitter receptor localization to postsynaptic specialization membrane |
| GO:0098696 | regulation of neurotransmitter receptor localization to postsynaptic specialization membrane |
| GO:0014827 | intestine smooth muscle contraction |
| GO:0015874 | norepinephrine transport |
| GO:0033033 | negative regulation of myeloid cell apoptotic process |
| GO:0036321 | ghrelin secretion |
| GO:0043568 | positive regulation of insulin-like growth factor receptor signaling pathway |
| GO:0045409 | negative regulation of interleukin-6 biosynthetic process |
| GO:0099170 | postsynaptic modulation of chemical synaptic transmission |
| GO:0099633 | protein localization to postsynaptic specialization membrane |
| GO:1904306 | positive regulation of gastro-intestinal system smooth muscle contraction |
| GO:1905564 | positive regulation of vascular endothelial cell proliferation |
| GO:1990314 | cellular response to insulin-like growth factor stimulus |
| GO:1990770 | small intestine smooth muscle contraction |
| GO:0030252 | growth hormone secretion |
| GO:0042536 | negative regulation of tumor necrosis factor biosynthetic process |
| GO:0060123 | regulation of growth hormone secretion |
| GO:1904304 | regulation of gastro-intestinal system smooth muscle contraction |
| GO:2000109 | regulation of macrophage apoptotic process |
| GO:1904347 | regulation of small intestine smooth muscle contraction |
| GO:1904349 | positive regulation of small intestine smooth muscle contraction |
| GO:1904468 | negative regulation of tumor necrosis factor secretion |
| GO:2000110 | negative regulation of macrophage apoptotic process |

**Supplementary Table 5**. Functional annotation of GHSR.

| **GeneOntology** | **Biological process** |
| --- | --- |
| GO:0042748 | circadian sleep/wake cycle. non-REM sleep |
| GO:0045187 | regulation of circadian sleep/wake cycle. sleep |
| GO:0045938 | positive regulation of circadian sleep/wake cycle. sleep |
| GO:0016520 | growth hormone-releasing hormone receptor activity |
| GO:0045188 | regulation of circadian sleep/wake cycle. non-REM sleep |
| GO:0046010 | positive regulation of circadian sleep/wake cycle. non-REM sleep |
| GO:0043568 | positive regulation of insulin-like growth factor receptor signaling pathway |
| GO:0021984 | adenohypophysis development |
| GO:0030252 | growth hormone secretion |
| GO:0060123 | regulation of growth hormone secretion |
| GO:0060124 | positive regulation of growth hormone secretion |
| GO:0060126 | somatotropin secreting cell differentiation |
| GO:0060133 | somatotropin secreting cell development |

**Supplementary Table 6.** GHRHR functional annotation.

| **GeneOntology** | **Biological process** |
| --- | --- |
| GO:0007190 | Аctivation of adenylate cyclase activity |
| GO:0002029 | Desensitization of G-protein coupled receptor protein signaling pathway |
| GO:0031018 | Endocrine pancreas development |
| GO:0032024 | Positive regulation of insulin secretion |
| GO:0048678 | Response to axon injury |
| GO:0070542 | Response to fatty acid |

**Supplementary Table 7.** GIPR functional annotation.

| **GeneOntology** | **Biological process** |
| --- | --- |
| GO:0007190 | Activation of adenylate cyclase activity |
| GO:0019933 | cAMP-mediated signaling |
| GO:0045777 | Positive regulation of blood pressure |
| GO:0071377 | Cellular response to glucagon stimulus |
| GO:0004967 | glucagon receptor activity |

**Supplementary Table 8.** GLP-1R functional annotation.

| **GeneOntology** | **Biological process** |
| --- | --- |
| KEGG:04024 | cAMP signaling pathway |
| KEGG:04080 | neuroactive ligand-receptor interaction |
| REACTOME:18372 | Secretin family receptors class B/2 |
| REACTOME:18377 | Glucagon-type ligand receptors |
| REACTOME:19327 | G alpha (s) signalling events |
| REACTOME:21340 | GPCR ligand binding |

**Supplementary Table 9.** Signaling pathways of GIPR and GLP-1R involvement.
